# SLiMs prediction method based on enhanced attention mechanism and feature fusion

**DOI:** 10.1093/bioadv/vbaf240

**Published:** 2025-10-01

**Authors:** Yifan Hao, Hao He

**Affiliations:** Department of Communication Engineering, School of Electronic and Information Engineering, Hebei University of Technology, Tianjin 300401, China; Department of Communication Engineering, School of Electronic and Information Engineering, Hebei University of Technology, Tianjin 300401, China

## Abstract

**Motivation:**

Short linear motifs (SLiMs) are functional regions composed of short sequences of specific amino acids. They usually do not have independent 3D three-dimensional structures, but play important roles in biological processes. Traditional detection methods have high cost and heavy workload, therefore it is necessary to seek an accurate detection method for SLiMs.

**Results:**

In this paper, we propose a new SLiMs prediction method, named EMAF_SLiMs, based on enhanced attention mechanism and feature fusion. We calculate three features sets which contain semantic embedding, physicochemical characteristic and evolutionary information. Then, we design the enhanced attention model based on SwiftFormer to highlight the characteristic of SLiMs. In addition, the multi-head attention mechanism is employed to effectively fuse these three feature sets. Finally, we construct an MLP network for prediction. EMAF_SLiMs has better performance on independent test sets, compared to other existing methods.

**Availability and implementation:**

The source code and sample data are available via a Github project at https://github.com/jdchhh/EMAF_SLiMs/tree/master.

## 1 Introduction

Short linear motifs (SLiMs), also known as eukaryotic linear motifs, are short stretches of contiguous amino acids, typically 3–15 residues in length, encoding protein–protein interaction sites. They are mainly located in the intrinsically disordered regions (IDRs) of proteins and are typically found to be highly conserved in orthologous proteins (Davey *et al.* 2009, Glavina et al. 2022). SLiM-mediated interactions play an essential role in cellular processes and signaling networks, including the regulation of homeostasis, apoptosis, and differentiation (Davey *et al.* 2012b, Roey *et al.* 2014). Dysregulation of SLiM-dependent PPIs can drive tumorigenesis by aberrantly activating oncoproteins or disabling tumor suppressors (Fasano *et al.* 2022, Davey *et al.* 2023). Linear motifs (short peptide sequences) act as modular building blocks, enabling interactions with various partners and aberrant tau aggregation is linked to misregulated motif interactions (Cehlar *et al.* 2021). As a result, SLiM-mediated protein interactions emerge as viable targets for therapeutic intervention (Dobson *et al.* 2021, Simonetti *et al.* 2023).

At present, there are generally two methods to predict and identify SLiMs: experimental methods and computational methods. Laboratory researchers use lab-based methods such as X-ray spectroscopy (Receveur-Bréchot *et al.* 2006), nuclear magnetic resonance (NMR) (Konrat 2014) to identify them in proteins. However, their short length (Semenza *et al.* 1990, Koch *et al.* 1991) poses great challenges for detection. And these traditional wet-lab methods are labor-intensive, too costly, time-consuming, and not suitable for large-scale implementation in commercial, biological, and medical fields. Among the computational methods, there are two main categories of methods. The first class of methods relies on regular expressions to identify SLiMs in protein sequences. For example, SLiMFinder (Edwards *et al.* 2007), QSLiMFinder (Palopoli *et al.* 2015), SLiMProb (Davey *et al.* 2010), SLiMSearch2.0 (Davey *et al.* 2011), SLiMSearch4.0 (Krystkowiak and Davey 2017), and SliMPrints (Davey *et al.* 2012a). Although the regular expression method combined with multidimensional information has made progress in SLiMs prediction, its core bottleneck lies in the reliance on sequence context, data quality, computational cost, and functional verification, which urgently needs to be further optimized by introducing techniques such as machine learning, dynamic modeling, or high-throughput experimental verification. The second computational method utilizes machine learning models to predict SLiMs, such as SLiMPred (Mooney *et al.* 2012) and PepBindPred (Khan *et al.* 2013). Both approaches employ bidirectional recurrent neural network models. Feature construction relies on hand-engineered derived features such as secondary structure and solvent accessibility, which is essentially a dimensionality reduction expression of sequence information and loses the high-order combination of physicochemical properties in the original sequence. Bidirectional recurrent neural networks can capture local context, but their ability to model ultra-long-range dependencies is limited. Although many computational methods have been proposed, there are still certain limitations.

In this paper, we propose EMAF_SLiMs based on enhanced attention mechanism and feature fusion. EMAF_SLiMs integrates three heterogeneous features: semantic embedding, physicochemical characteristic, and evolutionary information. The first feature set Seqfea is obtained by the Prot-BERT embedding and PCA. The second feature set Phyfea is selected by simulated annealing algorithm from the AAindex. The third feature set is evolutionary information calculated by PSSM. We also design an enhanced attention model to extract features of SLiMs more effectively. Moreover, the multihead attention mechanism is employed to effectively fuse the three feature sets. Finally, an MLP network is constructed for prediction. The experimental results show that, the AUC value of EMAF_SLiMs reaches 0.8217, significantly higher than that of MEME and GLAM2. In addition, we also compare the FPR values under different TPR. The FPR value of EMAF_SLiMs is significantly lower than that of MEME and GLAM2.

## 2 Methods

### 2.1 Datasets

A commonly used database of short linear motifs is ELM (Puntervoll *et al.* 2003, Gould *et al.* 2010, Dinkel *et al.* 2012, 2014, 2016, Gouw *et al.* 2018, Kumar *et al.* 2020, 2022, 2024), which provides a comprehensive, regularly updated, well-organized, manually curated and experimentally validated library of short linear motifs. By extracting and analysing SLiMs regions within protein sequences, we construct a deep learning model aimed at predicting SLiMs residues. This model can help identify functionally important regions that may be involved in specific biological processes or diseases. To support model training, we built feature vectors based on the sequence context surrounding each residue. We selected 2612 non-redundant protein sequences from the ELM database. To prevent data leakage from sequence similarity (e.g. homologous proteins or overlapping motifs), we clustered sequences with CD-HIT (40% identity threshold) to group homologs without reducing total count. Using a strict whole-cluster assignment strategy (all sequences in a cluster assigned to one dataset), we split the data into training/validation/test sets at a 6:2:2 ratio based on cluster distribution, resulting in 1566, 523, and 523 sequences respectively. To balance the training data set, we randomly down-sampled the negative samples so that the number of residues SLiM and non-SLiM is equal. For validation and test datasets, we retain the original distribution to reflect real-world class imbalance. The results of the similarity analysis are presented in the supplementary materials, available as supplementary data at *Bioinformatics Advances* online. The datasets used in this paper are shown in (Table 1).

**Table 1.** Summary of all the datasets used in this paper.

Number	Protein sequences	SLiMs residues	Non-SLiMs residues
Training dataset	1566	17 618	17 618
Validation dataset	523	5716	391 639
Test dataset	523	5869	386 446

### 2.2 The architecture of EMAF_SLiMs

**Figure 1.**
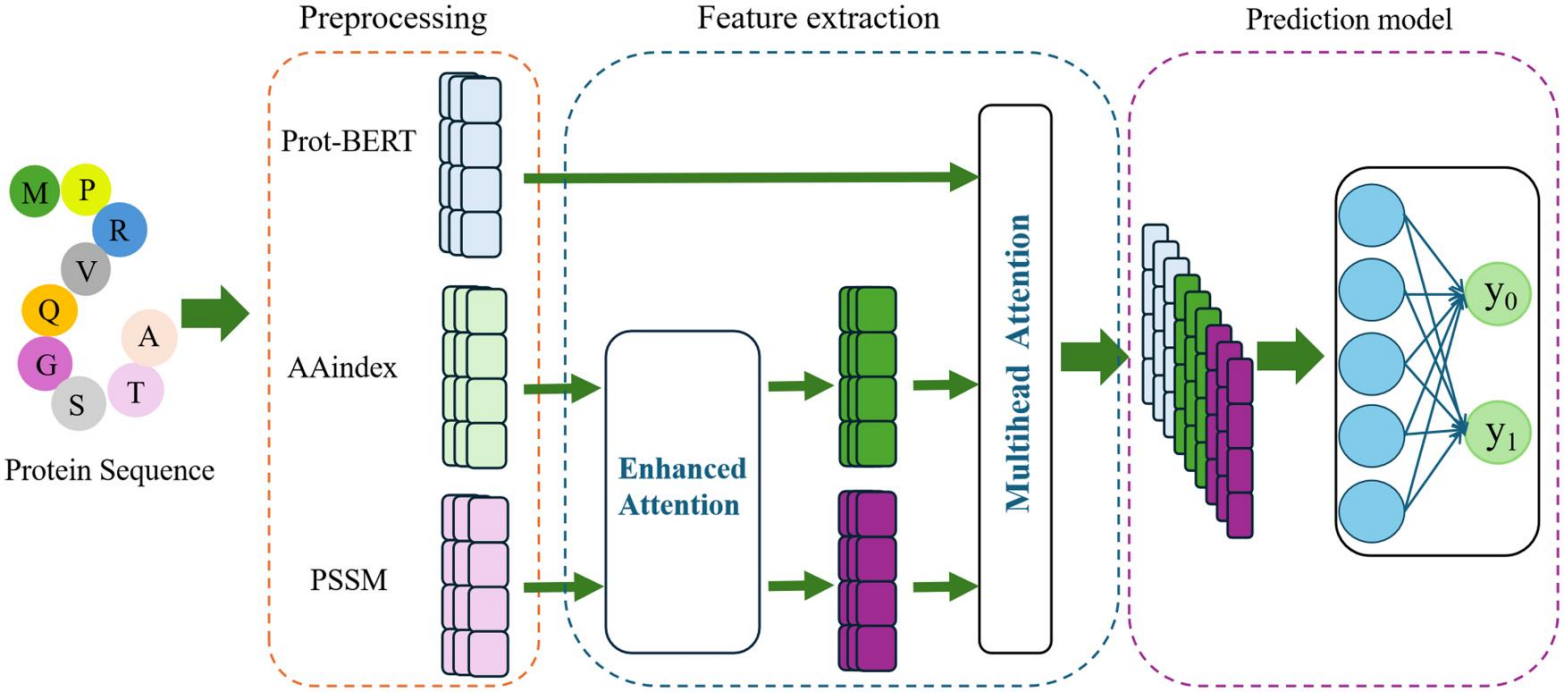
The architecture of EMAF_SLiMs.

The process architecture diagram of our method is shown in (Fig. 1). For a protein sequence, we preprocess it and calculate three characteristics including the characteristics embedded in the pre-trained model Prot-BERT, the physicochemical features from AAindex, and the PSSM matrix. Then we employ the enhanced attention mechanism in the feature extraction model to enhance the physicochemical features from the AA index and the PSSM matrix. After obtaining the output, the multi-head attention mechanism is used to further extract the characteristics of SLiMs residues from them and Prot-BERT. Finally, we employ the prediction model constructed based on MLP to train and predict the protein sequence to obtain the output results.

#### 2.2.1 Preprocessing

**Figure 2.**
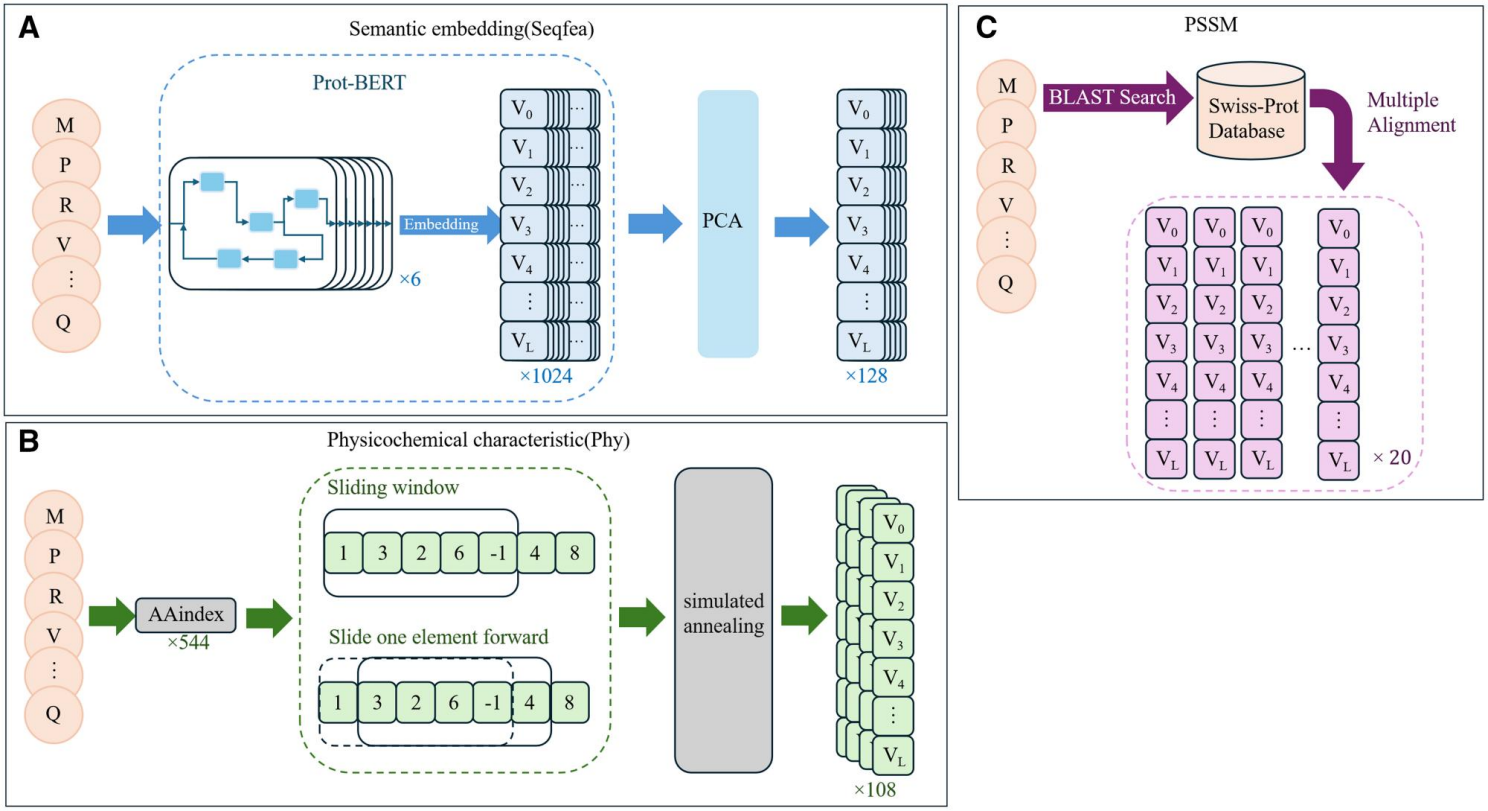
Preprocessing of protein sequences. (A) The calculation process of the semantic embedding. (B) The calculation process of physicochemical characteristic. (C) The calculation process of evolutionary information.

For each protein sequence, we calculate three kinds of feature sets: semantic embedding, physicochemical features and evolutionary information. We name these features as Seqfea, Phy, and PSSM, respectively, as shown in Fig. 2. As is shown in Fig. 2A, the first feature set, Seqfea, is obtained by Prot-BERT embedding (Lee *et al.* 2020, Ofer *et al.* 2021) and PCA. When using Prot-BERT embedding for feature extraction of protein sequences, if the output *L* × 1024-dimensional high-dimensional feature matrix is directly used for training, there is a significant computational burden problem. Moreover, the noise and redundant information commonly existing in the high-dimensional space will interfere with the model learning and reduce the generalization ability. For this purpose, principal component analysis (PCA) is adopted in the paper for feature dimension reduction. The original 1024-dimensional features are reduced to 128 dimensions. This dimension selection not only maximizes the original data information by retaining the principal components, but also reduces training complexity and noise interference, laying an efficient and robust feature foundation for subsequent training and prediction tasks.

As is shown in Fig. 2B, the feature set Phyfea is selected from AAindex (Kawashima and Kanehisa 2000) database by simulated annealing algorithm. There are 544 different features of amino acids in AAindex. First, 544 features are mapped, and then the average value is calculated twice by using sliding window. Finally, the simulated annealing algorithm is used to filter out the final features. We experimentally selected multiple sliding windows with *N* = 5, 7, 9, 11, 21, 41. The parameters of the simulated annealing are set as the initial temperature T0 = 100, the termination temperature T1 = 0.1, the annealing rate = 0.95, and the kernel function of SVM network is “linear.” In order to generate multiple sets of input feature matrices, we first obtain a 1 × 10 dimension and 1 × 20 dimension feature matrix for each protein sequence according to the optimal feature combination under each window. In addition, for multiple groups of optimal features of multiple Windows, 54 features with the highest frequency of feature index numbers were selected, and the window numbers with better evaluation results *N* = 7 and 11 were used for preprocessing. Finally, we obtain the L × 108-dimensional feature matrix of a protein sequence of length *L*.

As shown in Fig. 2C, the third feature set contains evolutionary information encoded by position-specific scoring matrix (PSSM) (Chowdhury *et al.* 2017). To construct this matrix, we use the PSI-BLAST tool (Altschul *et al.* 1998) to perform three rounds of iterative homology searches on the Swiss-Prot (Bairoch and Apweiler 2000) database. This database is a gold standard resource that has undergone strict manual annotation and sequence verification, and its data quality is widely recognized in proteomics research. During the search process, the *E*-value threshold is set at 0.001, which aims to balance the sensitivity and specificity of the search and conforms to the general standards of evolutionary proteomics research (Nie and Deng 2022). The three-round iterative strategy is chosen to ensure the convergence of PSSM construction (Pearson *et al.* 2017): each round of iteration would incorporate newly identified homologous sequences into the matrix update, thereby capturing subtle conserved patterns in the protein sequences. The advantages of the Swiss-Prot database include functional annotation, cross-referencing of experimental data, and redundant sequence filtering, ensuring that the obtained homologous sequences can reflect systematic evolutionary substitution features. These features are ultimately transformed into position-specific logarithmic probability scores in the PSSM, which quantify the evolutionary substitution probability at each residue position and provide a robust feature input for the subsequent prediction model construction. Finally, we obtain the *L* × 20-dimensional feature matrix of a protein sequence of length *L*.

#### 2.2.2 Feature extraction

**Figure 3.**
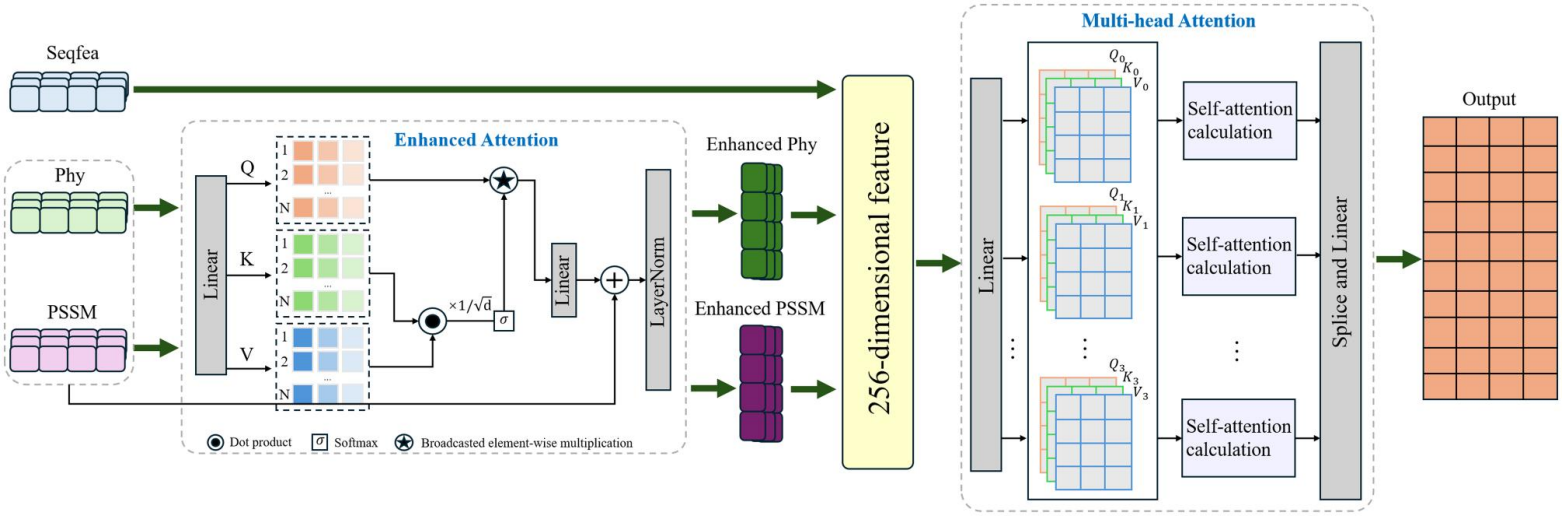
Feature extraction.

The calculation process of feature extraction is shown in Fig. 3. First, we take Phy and PSSM as the inputs of the enhanced attention model to obtain the enhanced output characteristics. Then, they are concatenated with the Seqfea features to obtain a 256-dimensional feature combination and input into the multihead attention mechanism for further feature extraction. EnhancedAttention based on SwiftFormer (Shaker *et al.* 2023) integrates residual connections and layer normalization within a single attention head, retains the original features and conducts stable training, avoiding the redundant computation of traditional multihead self-attention and achieving lightweight feature enhancement.

Moreover, the input and output dimensions are strictly consistent, adapting to the direct fusion of multimodal heterogeneous features. The input features are $X\in R^{B\times L\times D}$, where *B* is the batch size, *L* is the sequence length, and *D* is the feature dimension. The query matrix *Q*, key matrix *K* and value matrix *V* are generated by three linear layers, respectively, with the following formula (Equation 1),


(1) $$Q=X\cdot \;W_{Q},K=X\cdot \;W_{K},V=X\cdot \;W_{V}.$$

where $W_{Q},W_{K},W_{V}\in R^{D\times D}$ is the weight matrix of the linear layer. The attention score is calculated and scaled using the following formula,


(2) $$\text{Attention}\_\text{scores}=\frac{QK^{T}}{\sqrt{d_{k}}}.$$

where $d_{k}$ is the dimension of the key vector and $K^{T}$ is the transpose of the key matrix. The attention scores are normalized by softmax using the following formula,


(3) Attention_weights=softmax(Attention_scores).

We compute the attention output by element-wise multiplying the attention weights with the value matrix ***V***, which can be summarized as,


(4) Attention_output=Attention_weights *  V.

Here, * denotes the element-wise multiplication operation. After that we apply a linear projection to the attention output, which can be summarized as,


(5) $$\text{Projected}\_\text{output}=\text{Attention}\_\text{output}\;\cdot \;W_{o}.$$

where $W_{o}\in R^{D\times D}$ is the weight matrix of the linear layer. Finally, after residual connection and layer normalization, we obtain the enhanced output feature, which can be summarized as,


(6) Enhanced_output=LayerNorm(X+Projected_output).

After concatenating three types of features, namely Seqfea (128-dimensional, semantic embedding), enhanced PSSM (20-dimensional, evolutionary information), and enhanced Phy (108-dimensional, physicochemical characteristics), we obtain a 256-dimensional fused feature matrix. We extract the global dependencies of three types of features through four-head multihead attention, breaking through the limitations of simple splicing or linear fusion.

#### 2.2.3 Prediction model

**Figure 4.**
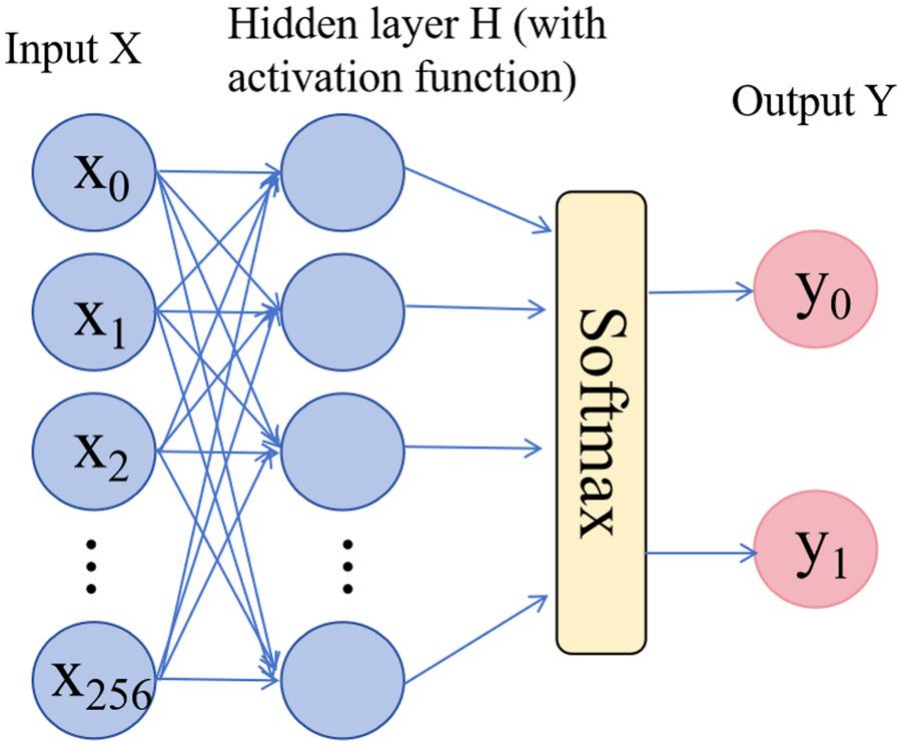
MLP network structure.

The neural network is built using the Python framework PyTorch v1.12. As is shown in Fig. 4, the MLP has 256 neurons in the input layer and 64 neurons in the hidden layer, using GELU as the activation function, followed by a dropout layer with a dropout rate of 0.3.

A second linear layer is used to map the features to the final classification output. The final output is a probability distribution for each class, calculated via a softmax activation function. Model Training and prediction: The Adam optimizer is used to train the classifier with the learning rate set to 0.001. The loss function used during training is the binary cross-entropy loss, which measures the difference between the predicted probability of the model and the true label.

## 3 Results

### 3.1 Evaluation metrics

In this paper, the objective is to predict whether each residue in the protein sequence belongs to a SLiMs residue or a non-SLiMs residue, which is a binary classification problem. We mainly utilize receiver operating characteristic (ROC) curve and the area under the ROC curve (AUC) as well as precision-recall (PR) curve and average precision (AP) to evaluate the performance. In addition, to evaluate the performance in detail, we also calculate the false-positive rate (FPR) at different the true-positive rate (TPR). The FPR and TPR can be denoted as:


(7) $$\text{TPR}=\frac{\text{TP}}{\text{TP}+\text{FN}}$$

(8) $$\text{FPR}=\frac{\text{FP}}{\text{TN}+\text{FP}}$$

where TP and TN represent the numbers of accurately predicted SLiMs and non-SLiMs residues, respectively. FP and FN represent the numbers of false positives and false negatives, respectively.

### 3.2 Performance comparison of feature processing with different window sizes

**Figure 5.**
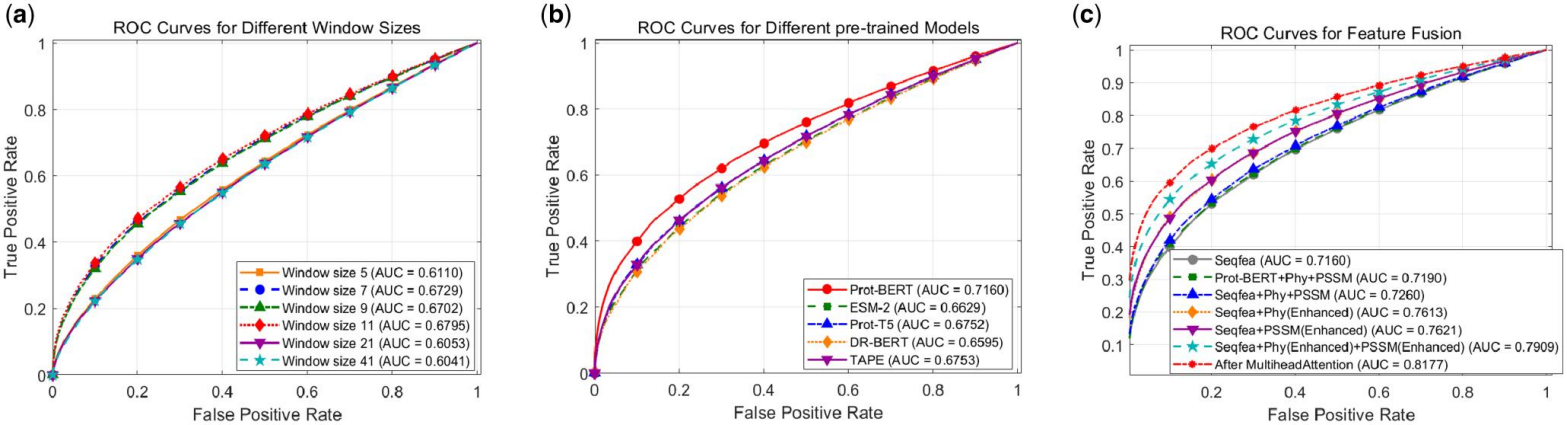
(A) Experimental results of window size ablation; (B) experimental results of pre-trained model embedding feature ablation; (C) experimental results of feature fusion method ablation.

As is shown in Fig. 5A, the AUC results of different window sizes on the validation dataset show that the AUC values of all window sizes are between 0.60 and 0.68, indicating that the overall performance of the classifiers corresponding to the Phy features is relatively close and superior to random guessing. Among them, the classifier with a window size of 11 has the best performance, an AUC value of 0.6795, the curve is closest to the upper left corner, and it has the strongest ability to recognize the balance of positive and negative classes. The classifiers with window sizes of 5 and 41 have poor performance, with AUC values of 0.6110 and 0.6041, respectively, and the curves are close to those of the random classifiers. The classifiers with window sizes of 7 and 9 have similar performances, and the AUC values are 0.6729 and 0.6702, respectively. The classifier with a window size of 21 also has poor performance, with an AUC value of 0.6053. This indicates that classifiers with smaller windows perform better in capturing local features, but windows that are too small or too large may lead to noise sensitivity or signal loss. To reduce the error, we chose Windows 11 and 7, but the effect still needs to be further improved.

### 3.3 Performance comparison of different pre-trained models

As is shown in Fig. 5B, we compare the performances of five pretrained models, TAPE (Rao *et al.* 2019), Prot-BERT (Lee *et al.* 2020), DR-BERT (Nambiar *et al.* 2024), Prot-T5 (Elnaggar *et al.* 2021), and ESM-2 (Lin *et al.* 2023) in the SLiMs prediction task. Prot-BERT ranks first with an AUC value of 0.7160, significantly ahead of 0.6629 of ESM-2, 0.6752 of Prot-T5, 0.6595 of DR-BERT, and 0.6753 of TAPE. This indicates that Prot-BERT has a more outstanding ability to represent high-dimensional features. Although there is a risk of overfitting in short sequence tasks, it can provide more comprehensive feature capture for SLiMs prediction. This comparison highlights the ability differences of different unsupervised BERT models in SLiMs prediction. The outstanding performance of Prot-BERT makes it a better choice for this task.

### 3.4 Performance comparison of feature fusion

As shown in Fig. 5C, from the comparison of AUC of different feature combinations, the AUC of using only Seqfea is 0.7160 and the AUC of Prot-BERT + Phy + PSSM is 0.7190 and the AUC of Seqfea + Phy + PSSM is 0.7260, and these results show that the overall performance of the feature combination is better than that of the single feature. Among them, the combination of features processed by PCA can improve AUC more effectively. To further explore the contribution of the two attention modules in the model, we conduct an ablation study. The AUC of Seqfea + Phy(Enhanced) is 0.7613. The AUC value of Seqfea + PSSM(Enhanced) is 0.7621. The AUC of Seqfea + Phy(Enhanced) + PSSM(Enhanced) reaches 0.7909. The AUC value of [Seqfea + Phy(Enhanced) + PSSM(Enhanced)]_MultiheadAttention skyrocket to 0.8177. Ablation studies confirm that the Enhanced Attention model enhances the quality of PSSM and Phy features, while the MultiheadAttention model refines the feature fusion and jointly improves the ability of the method to accurately predict SLiMs residues. The significant improvement in AUC emphasizes the effectiveness of the MultiheadAttention model in capturing complex feature interactions, verifies the key role of the MultiheadAttention model in multimodal feature fusion, and also shows the synergistic effect of the two attention mechanisms in improving the prediction performance of the method.

### 3.5 Performance comparison with other methods

To verify the effectiveness of EMAF_SLiMs, it is compared with MEME (Bailey *et al.* 2015) and GLAM2 (Frith *et al.* 2008) on the test dataset. MEME and GLAM2 are two well-known and widely used web server motif prediction tools.

**Figure 6.**
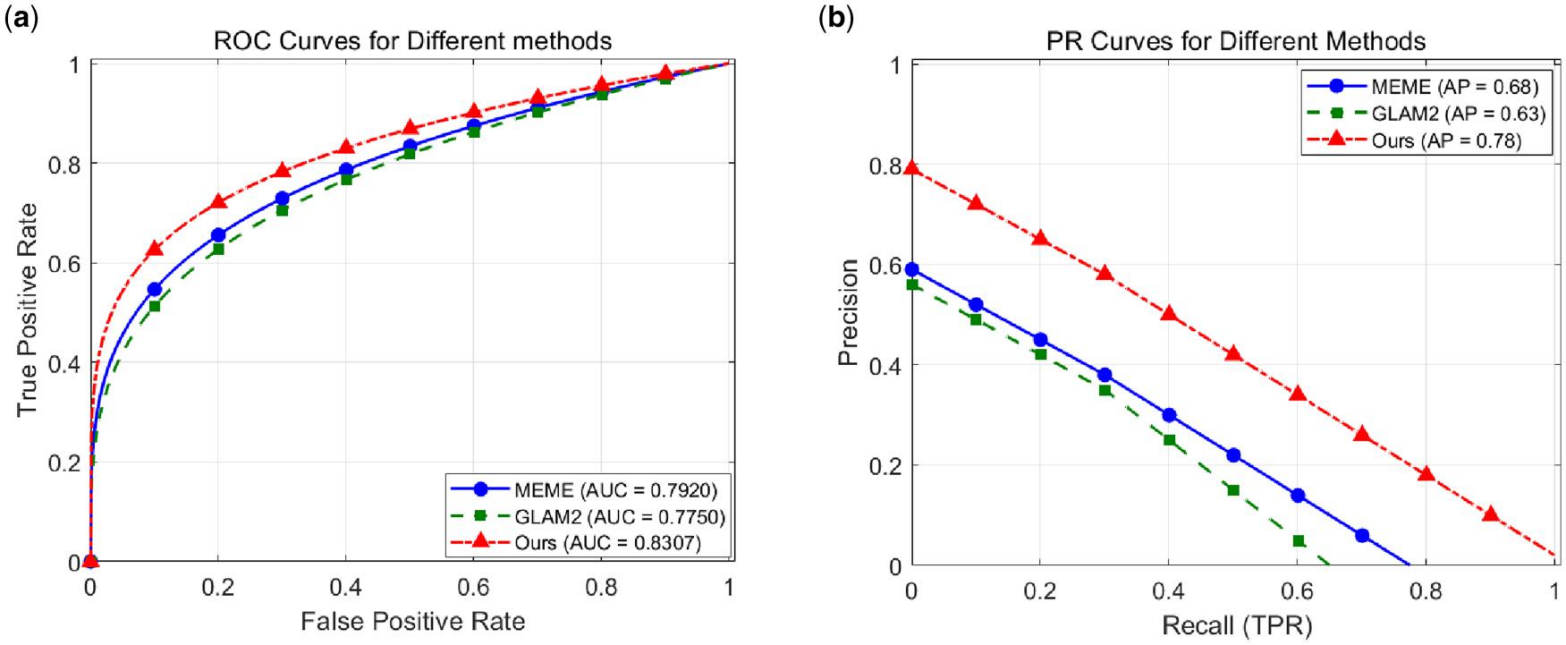
(A) Comparison of ROC results of the test set; (B) comparison of PR curve results of the test set.

As shown in Fig. 6A, in terms of AUC index, EMAF_SLiMs reaches 0.8217, which is significantly higher than 0.7840 of MEME and 0.7660 of GLAM2, indicating that its comprehensive ability to distinguish SLiMs residues and non-SLiMs residues is stronger. We also calculate the FPR values under different TPR to further analyse the performance of our method. As shown in Table 2, our EMAF_SLiMs obtains significantly lower FPR values than MEME and GLAM2. As shown in Fig. 6B, the PR curve and AP performance comparison on the test dataset further highlights the superiority of EMAF_SLiMs. With an AP of 0.78, EMAF_SLiMs outperforms MEME and GLAM2, indicating a better balance between precision and recall. As shown in Table 3, EMAF_SLiMs achieves higher precision than other methods under different TPR. These results show the advantage of EMAF_SLiMs in minimizing false positives and demonstrate its effectiveness and reliability in SLiMs prediction tasks.

**Table 2.** FPR (False Positive Rate) at different TPR (True Positive Rate) on the test dataset.

Methods/FPR	TPR = 0.2	TPR = 0.3	TPR = 0.4
MEME	0.0560	0.0412	0.1022
GLAM2	0.0520	0.0638	0.1319
**EMAF_SLiMs**	**0.0200**	**0.0223**	**0.0291**

The bolded values represent the lowest false positive rate among all the comparison methods under the corresponding TPR value.

**Table 3.** Precision at different TPR (True Positive Rate) on the test dataset.

Methods/Precision	TPR = 0.2	TPR = 0.3	TPR = 0.4
MEME	0.45	0.38	0.30
GLAM2	0.42	0.35	0.25
**EMAF_SLiMs**	**0.65**	**0.58**	**0.50**

The bolded values represent the highest precision value among all comparison methods under the corresponding TPR.

We use a simple test case example to illustrate the input format and the expected output, which can be found in the supplementary materials, available as supplementary data at *Bioinformatics Advances* online.

## 4 Discussion

The proposed method EMAF_SLiMs fuses three types of features: semantic embedding (Seqfea), physicochemical characteristic (Phy), and evolutionary information (PSSM). The Seqfea is obtained by the Prot-BERT embedding and PCA. The Phyfea is selected by simulated annealing algorithm from the AAindex database. The evolutionary information is calculated by constructing a position-specific scoring matrix (PSSM) using the PSI-BLAST tool based on the Swiss-Prot database. We introduce the enhanced attention model to enhance the key features of Phy and PSSM and improve the feature representation ability. In addition, we employ the four-head multihead attention mechanism to dynamically optimize the fusion matrix of simple stitching, addressing the limitations of single or simple stitching features in traditional methods and achieving effective feature fusion. The experimental results show that EMAF_SLiMs has significant advantages in modeling the complex feature relationships of protein sequences and identifying key functional fragments, proving the effectiveness and competitiveness of this method and providing new technical support for the functional research and mechanism analysis related to SLiMs.

The following points contribute to the good performance of EMAF_SLiMs. First, the three feature sets of protein sequences are effective for SLiMs prediction. Second, the preprocessing and feature extraction enhances the performance of these features. Third, the MLP-based prediction model constructed in this paper can capture the relationship between each feature and its neighboring features within the protein sequences feature matrix. This enables the method to extract richer information from different features, thus enriching the information provided by the protein sequence.

## 5 Conclusions

In this paper, we propose EMAF_SLiMs, a novel SLiMs prediction method based on enhanced attention mechanism and feature fusion. The experimental results show that, the AUC of EMAF_SLiMs on the independent testset reaches 0.8217, which is significantly higher than that of MEME and GLAM2. In addition, EMAF_SLiMs is able to obtain a lower FPR under the same TPR. For the extremely imbalanced data in this study, the AP index of EMAF_SLiMs on the independent testset reaches 0.78, which is significantly higher than the other two methods. This indicates that our method can more accurately reflect its ability to reduce false positives while capturing the SLiMs residues. EMAF_SLiMs is an efficient machine learning solution for SLiMs prediction, which lays a foundation for subsequent functional analysis and drug target mining. In the future, we can explore using deep learning networks to combine structured data predicted by AlphaFold to further improve prediction accuracy.

## Supplementary Material

vbaf240_Supplementary_Data
