# Supplementary material for: SLiMs prediction method based on enhanced attention mechanism and feature fusion
Source: Bioinform Adv. 2025 Oct 1;6(1):vbaf240. doi: 10.1093/bioadv/vbaf240 (PMC12782102; doi:10.1093/bioadv/vbaf240)
Supplement: vbaf240_Supplementary_Data [file vbaf240_supplementary_data.pdf]

supplementary materials

Analysis of Similarity Results

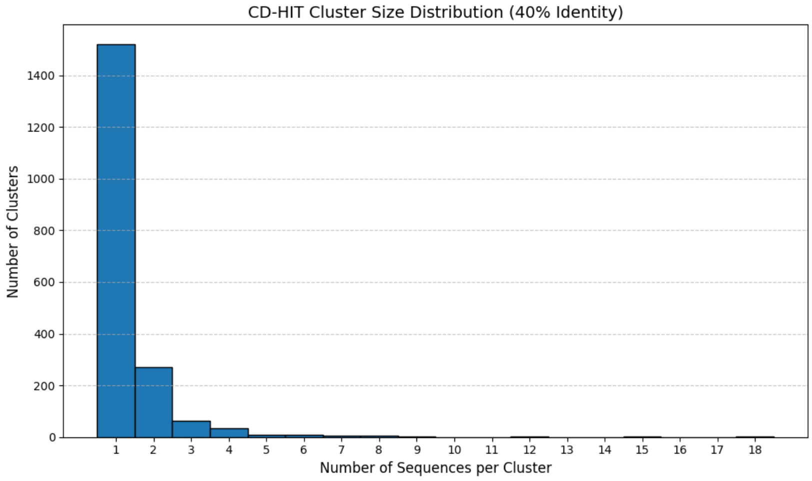

Supplementary Fig 1. Number of protein sequences in each homologous cluster.

The majority of clusters (over 1400) contain only 1 sequence, with cluster sizes ⩾2 being rare (e.g., ~250 clusters with 2 sequences, ⩽50 clusters for sizes ⩾3). This indicates that CD-HIT clustering effectively reduced redundancy, as most sequences are non-homologous (⩽40% identity) at this threshold. By assigning entire clusters to the same dataset (training/validation/test), we ensure no homologous sequences or overlapping SLiM regions are split across partitions, preventing data leakage and enhancing dataset independence.

An example demonstrating the input and output formats

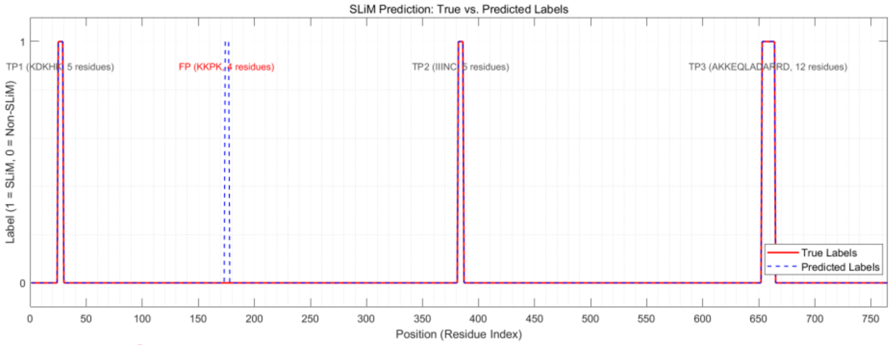

Supplementary Fig 2. Visualization of True Labels and Predicted Labels.

We chose the protein sequence P11387 from the testset. As shown in Supplementary Fig 2, there are three SLiMs regions in total, and under the condition of a threshold of 0.3, we have all correctly predicted them. For this sequence, the predicted TPR is 1.0 and the FPR is 0.00538. True label (red solid line): Represents the SLiMs regions verified by the experiment (TP1, TP2, TP3). Predicted label (blue dotted line): Displays the SLiMs regions predicted by the model, including true positives (TP) and false positives (FP).

Key region analysis：

1. True positives (TP): TP1 (KDKHK, 5 residues, 25 - 29): The red line and the blue line are completely overlapping, indicating accurate prediction. TP2 (IIINC, 5 residues, 382 - 386): Completely aligned, indicating that the model can recognize short SLiM motifs. TP3 (AKKEQLADARRD, 12 residues, 653 - 664): Completely overlapping, highlighting the effective detection of longer and more complex SLiMs sequences.
2. False positives (FP): FP (KKPK, 4 residues, 174 - 177): Blue dotted line but no red solid line, indicating a prediction error (regions that are not SLiMs are mistakenly classified as SLiMs).
